# The role and therapeutic potential of gut microbiome in severe burn

**DOI:** 10.3389/fcimb.2022.974259

**Published:** 2022-11-17

**Authors:** Zhijie Huang, Yisheng Huang, Jun Chen, Zhengming Tang, Yuanxin Chen, Hongyu Liu, Mingshu Huang, Ling Qing, Li Li, Qin Wang, Bo Jia

**Affiliations:** Department of Oral Surgery, Stomatological Hospital, Southern Medical University, Guangzhou, China

**Keywords:** severe burn, gut microbiome, intestinal mucosal barrier, microbial therapy, dysbiosis

## Abstract

Severe burn is a serious acute trauma that can lead to significant complications such as sepsis, multiple organ failure, and high mortality worldwide. The gut microbiome, the largest microbial reservoir in the human body, plays a significant role in this pathogenic process. Intestinal dysbiosis and disruption of the intestinal mucosal barrier are common after severe burn, leading to bacterial translocation to the bloodstream and other organs of the body, which is associated with many subsequent severe complications. The progression of some intestinal diseases can be improved by modulating the composition of gut microbiota and the levels of its metabolites, which also provides a promising direction for post-burn treatment. In this article, we summarised the studies describing changes in the gut microbiome after severe burn, as well as changes in the function of the intestinal mucosal barrier. Additionally, we presented the potential and challenges of microbial therapy, which may provide microbial therapy strategies for severe burn.

## Highlights

- Review of the composition of the normal gut microbiome and its changes after burns.
- Effects of the gut microbiome on the intestinal mucosal barrier after burns.
- Strategies and potential for microbial therapy of severe burns.

## Introduction

Burn injury is a kind of heat-induced damage to skin or mucus, which can be caused by various factors. Direct or indirect contact with high temperature, electric current, cold objects, and corrosive or highly radioactive substances can induce burn injury. Generally, most burn injuries are caused by thermal injuries, including flame, hydrothermal factors, high-temperature gas, laser, hot metal liquids or solids.

**Figure 1:**
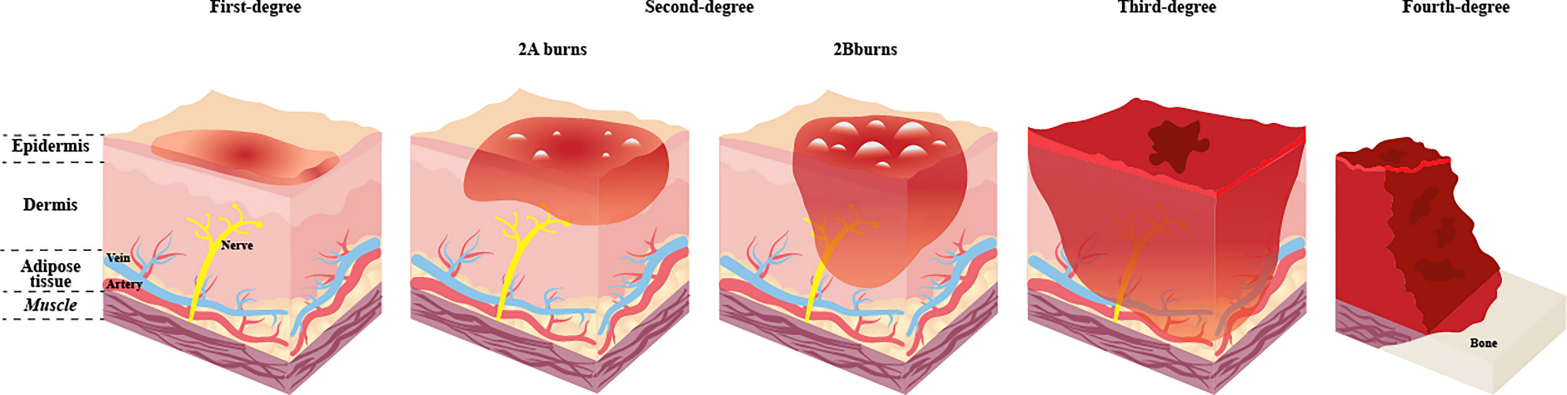
Categorization and depth of burn. The depth of the wound is significant for the categorization, treatment and prognosis of the burn. The deeper the burn, the more likely it is to operate and the more challenging it is to deal with postoperative scars. First-degree burns affect the epidermis only, with obvious pain, generally do not leave scars after healing, and no surgery is needed. Once the burns extend to the dermis, the injuries are classified as second-degree burns, blisters will form on the surface of the skin, and the pain is obvious, which can be divided into 2A burns and 2B burns. 2A burns do not require surgery and may leave scars. 2B burns require surgery and leave more scars. Third-degree burns affect the whole skin and subcutaneous adipose tissue. Fourth-degree burns involve damage to deep tissues such as muscles or bones, and defects and blackening are often seen at the burn site. Third-degree and fourth-degree burns have no significant pain due to damage to nerve endings and have a high risk of infection. Both need surgery.

The burn injury severity depends on the wound’s depth and size, and the proper assessment helps guide immediate treatment decisions. The burns’ depth can be divided into first, second, third, and fourth degree (**Figure 1**). In terms of size, it can be calculated according to the percentage of burn area to the total surface area (TBSA). And the “rule of nines” is commonly used to assess the proportion of TBSA. Burns can be classified as minor burn or major burn. Minor burn often involves less than 10% of TBSA and is mainly superficial. By contrast, Major burn is inconclusive in the burn size and is not well-defined. Older adults are more likely to have serious complications over a similar burn size than younger adults. On the other hand, children, whose body surface area is much smaller than adults, often have a more significant percentage of the burn area when burns occur. Therefore, the TBSA of severe burns is not consistent for burn patients of different ages. The current guidance for classifying severe burn injuries is as follows, greater than 20% TBSA in adults, greater than 10% TBSA in the elderly, and greater than 30% TBSA in children (Jeschke et al., 2020). Generally, an immediate inflammatory response is triggered to promote tissue healing after a minor burn (Stanojcic et al., 2018). However, the inflammatory response triggered by severe burns is different and unique. It is widespread and uncontrolled, further enhancing the inflammatory response and causing the body enters a systemic catabolic state, referred to as the hypermetabolic response. The hypermetabolic response is almost unique to major burn, often results in difficulty entering the healing phase, delayed wound healing, and is associated with increased rates of infection, organ failure and even death (Porter et al., 2016). Also, the hypermetabolic state can persist for up to one year after the burn (Porter et al., 2016). Therefore, early treatment of hypermetabolic states is essential. The most commonly used method in clinical practice is nutritional support therapy. The European Society for Clinical Nutrition and Metabolism (ESPEN) recommends 1.5-2.0 g/kg per day of protein intake for adults and 1.5-3.0 g/kg per day for children. The ratio of carbohydrates to the total energy intake is less than 60%, the speed is less than 5.0mg/kg per min, and the total fat intake is less than 35% of the total energy intake (Rousseau et al., 2013). These have guiding significance for clinical individualised nutritional support.

Severe burn injuries often destroy the local or systemic skin barrier, resulting in a local or life-threatening systemic infection. Previous studies have revealed that sepsis and infectious complications are the causes of death in most patients with severe burn (Church et al., 2006; Lachiewicz et al., 2017). Severe burn can cause direct damage to burning sites in the early stage, followed by a series of systemic responses, leading to catastrophic consequences such as sepsis, shock and multiple organ failure. Meanwhile, the cascade of systemic response will significantly affect the prognosis of patients. These systemic reactions are promoted by the conjunction action of many inflammatory factors and cells. After major burn, the capillary permeability increases, and large amounts of tissue fluid are lost to the extravascular space, leading to tissue oedema and decreasing cardiac output and effective circulating blood volume, leading to shock (Rae et al., 2016). Meanwhile, the body releases massive amounts of cortisone, catecholamines, and inflammatory cytokines, such as interleukin-6 (IL-6), IL-10 and tumour necrosis factor (TNF), into the systemic circulation, triggering a severe inflammatory reaction, runs out the immune stress and reduces systemic immunocompetence, leading the body into the susceptible state. Skin and mucosal barrier destruction increase the opportunity for pathogenic invasion. A large damaged area makes it easier for microorganisms to invade the blood circulation, and the wound can absorb ‘toxins’ back into the body during the aqueous resorption phase. Consequently, the body reaches the peak of systemic infection (Plichta et al., 2017). The intestinal mucosal barrier can cause stress damage after burns, which leads to the translocation of intestinal microorganisms and endotoxins, eventually becoming an essential source of endogenous infection (Rudd et al., 2020). In addition, burns are often accompanied by other damage such as inhalation injury or fracture, which are promoting factors that further weaken the body’s resistance and increase the risk to the life (Rehberg et al., 2009; Grigorian et al., 2018).

As the largest microbial reservoir of the body, the gastrointestinal tract contains approximately 1800 species of bacteria, mainly from the *Firmicutes* and *Bacteroidetes* phyla, as well as *Actinobacteria* and *Proteobacteria*, with a total of up to 100 trillion, forming a complex and dynamically balanced microecosystem (Meng et al., 2018; Durack and Lynch, 2019; Ruan et al., 2020). Normally, the gastrointestinal microbiome helps maintain human health. With the disruption of the dynamic balance of the microbial system, the microorganisms that initially help to preserve health may convert into pathogens that endanger the host. After severe burn, the mesenteric vasoconstriction creates a local hypoxic environment, resulting in changes in intestinal oxygen partial pressure, slowed intestinal peristalsis, and reduced mucus secretion. Thereby, the homeostasis of the intestinal microenvironment is disrupted, resulting in abnormal changes in the type, quantity, proportion and location of the intestinal microbiome (Huang et al., 2017; He et al., 2019; Guo et al., 2021). The subsequent ischaemia–reperfusion injury further aggravates intestinal mucosa damage, leading to the activation of cellular stress response and cell necrosis, which eventually damage the gut barrier, increasing the intestinal permeability and leading to the translocation of bacteria to mesenteric lymph nodes (Jones et al., 1990; Tadros et al., 2003; Magnotti and Deitch, 2005). Furthermore, medical interventions for patients with burns, such as antibiotics, nutritional changes and surgery, aggravate the impaired intestinal microecology and promote deterioration (Lachiewicz et al., 2017). For the past few years, given the widespread application of microbiology, metabolomics and genomics in researching the relationship between intestinal microorganisms and diseases, we have further advanced the understanding of the relationship between intestinal microorganisms and the development, treatment and healing of severe burn injuries. Therefore, in this review, we aimed to collect and summarise studies on intestinal microbiome after severe burn to explain the changes in intestinal microorganisms and the role of these changes in intestinal mucosal barrier destruction. In addition, we discussed the potential of some gut microbiome-based therapeutic strategies.

## Intestinal microbiome

### Normal intestinal microorganisms

The human microbiota is a complex and diverse group of bacteria, viruses, fungi, and other microbe colonised in specific body parts, and they are closely related to human health. Microorganisms begin to colonise the human body during fetal life, and a mature microbiota is developed within three years after birth, similar to that of adults (Yatsunenko et al., 2012; Mishra et al., 2021). The human microbes are mainly distributed in the skin, oral mucosa and gastrointestinal mucosa, in which the gastrointestinal tract is the largest microbial reservoir (Liu et al., 2021). Studies have indicated that the intestinal tract contains hundreds of trillions of bacteria that perform critical physiological functions, such as nutrient absorption, metabolism, immune system development and maturation, and pathogen colonisation (Wright et al., 2013). The microorganisms that live in the intestinal tract are numerous and diverse. Previous studies have reported that approximately 1000 individual bacterial species are colonised in the intestinal tract (Backhed et al., 2004). Latest studies have reported far more than 1000 species of the human intestinal microbiome, and there may be more than 18,000 (Eckburg et al., 2005; Ley et al., 2006). Most of these data are based on *in vitro* cultivation, and most of the bacteria in the intestinal tract cannot be cultured yet. Therefore, the actual number of bacteria in the intestinal tract will far exceed the current results. In recent years, with the development of 16S rRNA-sequencing technology, researchers can accurately distinguish microbial genus by sequencing the hypervariable region of the gene. Moreover, metagenomic next-generation sequencing (mNGS) can detect known and unknown microorganisms in samples without bias and analyse the genes of pathogens. Overall, the high-throughput sequencing technology can further deepen our understanding of the human microbiome in health and disease.

**Figure 2:**
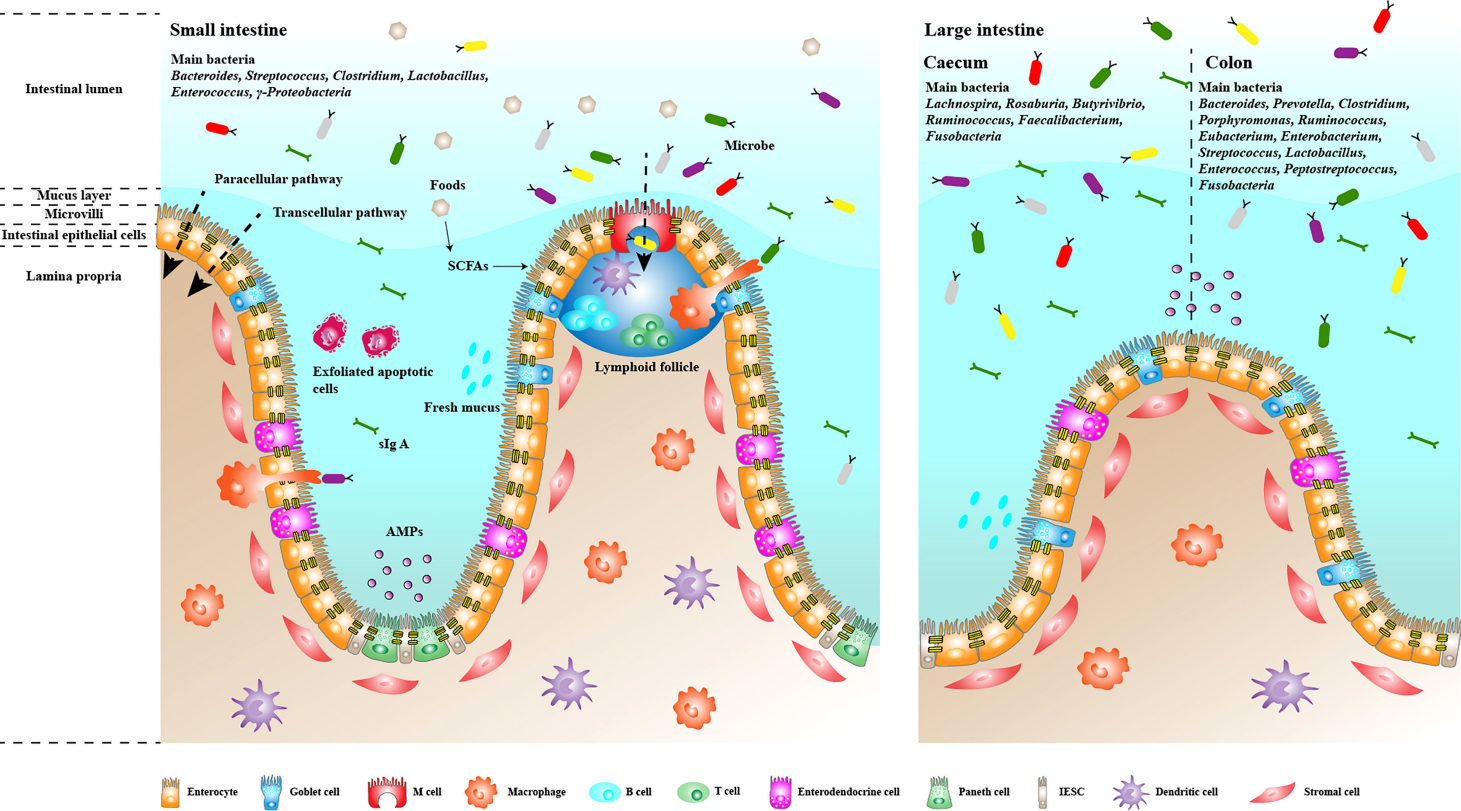
Composition and function of the intestinal mucosal barrier. The intestinal mucosal barrier has multiple mechanisms that help regulate intestinal homeostasis and prevent the invasion of pathogenic microorganisms and their metabolites in the intestinal lumen. Single layers of epithelial cells are bound together by apical junctions, constituting the most basic and essential barrier. The barrier regulates the transport of bacteria and antigens through the paracellular and transcellular pathways. Parts of epithelial cells, such as Goblet cells and Paneth cells, can secrete mucins and antimicrobial peptides to form the mucus layer and prevent the excessive growth and invasion of flora. M cells continuously take up intestinal antigens and induce subsequent mucosal immune responses. Plasma cells in the lamina propria secrete IgA into the intestine to form an immune barrier that prevents bacterial adhesion.

The human intestinal microbiota is a diverse population with a significant differences between individuals. Furthermore, people living in the same area and environment exhibit different microbial communities (Yatsunenko et al., 2012). Environmental factors (such as region and diet), individual genetics and host physiological conditions (such as sex, age, disease and obesity) all affect the structure of the bacterial community (Human Microbiome Project Consortium, 2012). In addition, the composition and abundance of microbial communities at different sites in the gastrointestinal tract vary greatly, except that the distal small intestine, colon, and large intestine share some commonalities (Vasapolli et al., 2019). Nonetheless, some gut microbiome studies have identified several bacterial phyla and associated genera. *Firmicutes*, *Bacteroidetes*, *Actinobacteria* and *Proteobacteria* phylum are the main intestinal microorganisms. *Firmicutes* and *Bacteroidetes* are the dominant flora. Most of them are obligate anaerobes, which significantly affect the function of the whole flora, and determine the physiological and pathological significance of the flora to the host. The secondary microbiome comprises aerobic or facultative anaerobes, such as *Escherichia coli* and *Streptococcus*, which are highly mobile, potentially pathogenic, opportunistic bacteria. The abundance of pathogenic bacteria species, such as *E. coli* and *Salmonella*, is relatively low, accounting for less than 0.1% of the total bacteria, generally entering the intestinal tract by accidental ingestion, which can cause diarrhoea and poisoning (Jandhyala et al., 2015; Ruan et al., 2020). Despite the lack of significant difference in the overall composition of the intestinal microbiome among humans, significant differences were found in the structure of the bacterial community in different regions of the intestinal tract (**Figure 2**) (O’Hara and Shanahan, 2006). The main microbiome of the small intestines includes *Bacteroides*, *Streptococcus*, *Clostridium*, *Lactobacillus*, *Enterococcus* and *γ-Proteobacteria*, with *Streptococcus* as the dominant genus. The main bacteria in the caecum are *Lachnospira*, *Rosaburia*, *Butyrivibrio*, *Ruminococcus*, *Faecalibacterium* and *Fusobacteria*. The main bacteria in the colon are *Bacteroides*, *Prevotella*, *Clostridium*, *Porphyromonas*, *Ruminococcus*, *Eubacterium*, *Enterobacterium*, *Streptococcus*, *Lactobacillus*, *Enterococcus*, *Peptostreptococcus* and *Fusobacteria* (Justesen et al., 1984; Pei et al., 2004; Jandhyala et al., 2015). A healthy gut microbiome presents high diversity. When microbial diversity decreases, the organism becomes more susceptible to various diseases, such as inflammatory bowel disease (IBD), obesity and colon cancer (Bisgaard et al., 2011; Karlsson et al., 2013; Ferreira et al., 2014). Some studies have reported that more *Firmicutes* than *Bacteroidetes* in the intestine lead to more effective absorption of calories in food, resulting in obesity, which in turn affects the susceptibility to diseases, indicating that the ratio of *Firmicutes* to *Bacteroidetes* is related to disease susceptibility (Ley et al., 2006; Le Chatelier et al., 2013).

The intestinal microbiome maintains a symbiotic relationship with the intestinal mucosa and confers healthy people with essential metabolic, immune and intestinal protective functions. In an experiment comparing the intestinal functions of germfree mice with that of normal mice, germfree mice presented severe defects in mucosal immune function, deficient production of cytokines, decreased intestinal motility and vascularity and reduced renewal rate of intestinal epithelial cells due to the absence of resident microflora (Shanahan, 2002). Intestinal microbiota obtains nutrients from the dietary components of the host and the exfoliated epithelial cells. It has extensive metabolic capacity and robust functional plasticity, equivalent to an organ of the human body. By fermenting the non-digestible carbohydrates and some xenobiotic substances, the intestinal microbes synthesise metabolites, a vital energy source for the cells throughout the body. Moreover, the metabolites enter the bloodstream, affecting the overall metabolism and weight, even insulin sensitivity (Turnbaugh et al., 2008; Pedersen et al., 2016). Among the metabolites, short-chain fatty acids (SCFAs) are the most extensively studied, including acetate, propionate and butyrate. Acetate and propionate are usually transported to the liver and other peripheral tissues for metabolism and converted into glucose or lipids. Butyrate is mainly transported to intestinal epithelial cells as its primary energy source (up to 80%) (Roediger, 1982; Bergman, 1990; Macfarlane and Macfarlane, 2011). In addition, butyrate is an anti-inflammatory molecule that can maintain intestinal immune homeostasis by promoting Th1 cell IL-10 production (Sun et al., 2018). Most bacteria in the intestinal tract can produce SCFAs, among which *Bifidobacterium* is widely studied for its ability to synthesise butyrate. Other butyrate-producing bacteria include *Faecalibacterium prausnitzii*, *Lachnospira*, *Anaerostipes* and *Eubacterium* (Louis and Flint, 2009; Riviere et al., 2016; Parada et al., 2019). On the other hand, SCFAs can regulate energy balance by interacting with receptor ligands of G protein-coupled receptor Gpr41 and promote intestinal barrier function through various mechanisms (Samuel et al., 2008). Some studies have found that SCFA therapy can promote the proliferation of epithelial cells, increase tight junctions (TJs) and reduce epithelial permeability (Peng et al., 2009; Barko et al., 2018). Furthermore, studies have presented that SCFAs can promote IgA production in the intestine, regulate the immune response of intestinal mucosa and provide antibacterial protection for the body (Wu et al., 2017). Therefore, maintaining a healthy intestinal microbiome is vital to maintain the normal functioning of the intestines.

### Changes and effects of the intestinal microbiome following severe burn

As the ‘hidden organ’ of the body, the intestinal microbiome can self-regulate and cope with the changes and challenges brought about by environmental factors and age, such as antibiotics and aging (Lozupone et al., 2012). When these factors exceed the regulatory capacity of the intestinal microbiome, the dynamic balance between intestinal microorganisms is disrupted, triggering the development of various diseases. The dynamic imbalance of the intestinal microbiome is generally called bacterial dysbiosis, which can be characterised by decreased intestinal bacterial diversity and increased pathogen abundance. It is closely related to inflammatory gastrointestinal disorders, such as inflammatory bowel disease (IBD) (Barko et al., 2018). Studies have revealed that compared with healthy people, patients with IBD have lower intestinal bacterial diversity, fewer beneficial bacteria and more *Enterobacterium* such as *Proteobacteria* (Halfvarson et al., 2017). These changes can affect both the progression and prognosis of the disease. Severe burn is an acute trauma significantly different from IBD. However, intestinal inflammation and destruction of the intestinal mucosal barrier are common manifestations which may be closely related to bacterial dysbiosis. Therefore, the role of gut bacteria in burns is as important, or even more prominent, as in chronic inflammatory diseases.

Previous studies have focused on the changes in intestinal microbiome composition following severe burn. Earley et al. (2015) found that *Bacteroidaceae*, *Lachnospiraceae* and *Ruminococcaceae* families were the dominant flora in the control group. In contrast, *Bacteroidaceae* and *Ruminococcaceae* families dramatically decreased in patients with burns, with a change in the composition, by comparing the abundance of bacteria in the faecal samples. In addition, the abundance of γ-*Proteobacteria* increased markedly, especially those in the *Enterobacteriaceae* family. The *Enterobacteriaceae* family contains many opportunistic pathogenic bacteria common in patients with bacterial translocation and sepsis, such as the genera *Escherichia*, *Proteus*, *Klebsiella*, and *Citrobacter* (MacFie et al., 1999). Hence, the increase in the *Enterobacteriaceae* family, especially strains of *Escherichia*, can provide a promising target for treating severe burn. Wang et al. (2017) studied the dynamic changes in the gut microbiome in severely burned patients during the whole course of the disease. The results showed that the intestinal flora began to appear imbalanced in the early stage of severe burn (from 2-4 weeks). The microbial diversity decreased significantly while the balance was gradually restored. The microbial diversity gradually returned to the average level at the later stage. Additionally, in the early stage, the beneficial genus, such as *Bacteroides*, decreased dramatically, while the opportunistic pathogen genus *Enterococcus* and *Escherichia* became the majority intestinal microbiome. And the *Bacteroides* gradually regained the dominant position in the gut flora at the end of the study. The above studies both showed that the microbiome shift in severely burned patients is seen similarly in patients with gastrointestinal inflammation, such as IBD (Frank et al., 2007). Interestingly, Lima et al. (2021) reported conflicting results with Wang in a longitudinal analysis of cutaneous and gastrointestinal microbiota patients with severe burn. They found that the microbial profile of representative intestinal samples (rectal and perianal) did change over time, but there was no significant difference in species numbers and evenness. The results of this contradiction may be related to the fact that all the patients with severe burn in this study were male and that the sample size was small (ten patients). It shows that increasing the sample size is necessary to deeply understand the changes in intestinal microflora in patients with severe burn. Pan et al. (2020) investigated the characteristics of intestinal microbiome dynamic changes after burn and its relationship with different stages of burn course by detecting the intestinal flora of patients with severe burns during hospitalisation. At the phylum level, the relative abundance of intestinal flora in patients in the shock stage (within three days after injury) was the highest in *Firmicutes*, *Proteobacteria* and *Bacteroidetes*. In the acute infection stage, the relative abundance of *Firmicutes* decreased, whereas that of *Proteobacteria* and *Bacteroidetes* increased. At the family level, the distribution of microflora in patients in the shock stage was more uniform, and the top five dominant bacteria were *Enterococcaceae*, *Ruminococcus*, *Lactobacillaceae*, *Spirillaceae* and *Enterobacteriaceae*. During the acute infection stage, *Enterobacteriaceae*, *Bacteroidaceae* and *Streptococcaceae* were the prominent families. However, the abundance of *Ruminococcus*, *Lactobacillaceae* and *Spirillaceae* decreased sharply, most of which were SCFA-producing bacteria.

In another way, Changes in metabolite concentration often accompany the change in intestinal microbiota. Shimizu et al. (2015) measured the fecal samples from patients with severe burn. It was found that the abundance of beneficial bacteria decreased significantly in the early stage after burn, especially *Bifidobacterium*, which was the main butyrate-producing bacteria. Further assession of the concentration of SCFAs in fecal samples showed that acetate, butyrate and propionate levels decreased in patients with severe burns. These changes recovered to the average level in the late stage of burn. However, the levels of butyric acid, propionic acid, valeric acid and isobutyric acid remained undetectable in the only death in this study. Pan et al. (2020) analysed the functional changes in the intestinal flora after severe burn and found that the relative abundance of functional genes, such as those involved in cysteine and methionine metabolism, glycolysis and gluconeogenesis, pyruvate metabolism and ribosomal synthesis decreased in the early stage of acute infection (4-14 days after injury). However, the relative abundance of functional genes, such as those involved in amino acid and sugar metabolism, transport and peptidase, peaked at the middle stage (15-28 days after injury) and late stage (from 29 days after injury to 1 week before discharge) of infection. These results indicate that in the early and middle stages of acute infection, some functions related to amino acid metabolism, glycolysis, gluconeogenesis and pyruvate metabolism of intestinal flora are weakened, which leads to a decrease in SCFAs synthesis. The concentration of SCFAs in the intestinal cavity and pH level decreased. SCFAs have protective effects on the intestinal mucosa, and intestinal pH indicates an intestinal flora disorder. These changes reflect the significant impact of intestinal microbiota changes on disease progression in patients with severe burn. Moreover, patients usually experience weeks or even months of hypermetabolism response after severe burn. Therefore, it is necessary to provide appropriate, timely and adequate nutritional support in the early stage. Although only five samples were included in this study, it gives a reference for guiding the timing and provision of nutritional support to patients with severe burn. The above human studies show us some cognition and indication of the changes in gut microbiome after severe burn, but the sample size is small. Therefore, further study is needed to explore the changes in the microbe and metabolites in patients with severe burns.

Various confounding factors, such as antibiotics and primary diseases, can affect the clinical samples. Therefore, animal experiments are needed to control these confounding factors to ensure the reliability of the research results. The animals commonly used to establish burn models are mice, rats and pigs. As an animal research model, murine has many advantages. Murine has a large family size and a formal pedigree structure. It is easy to measure phenotypic parameters and can stably construct a large number of models. Meanwhile, murine-specific reagents are diverse, and murine is sensitive to transgenic technology, which helps researchers to provide useful insights into the signal pathways involved in the disease development and healing process. In addition, murine has a superior immune system and wound healing is very fast, which are helpful for researchers to study the mechanism of wound healing more quickly (Mestas and Hughes, 2004; Wong et al., 2011). However, the relatedness between murine and human is far away, and there are great differences in skin structure and immune system. Additionally, the murine burn model’s metabolic characteristics are very different from those of humans (Caldwell et al., 1959; Abdullahi et al., 2014). Hence, these factors make it difficult to directly translate the research results of the murine burn model into clinical application. The physiological function and anatomical structure of pigs are similar to those of humans, especially the biochemical characteristics and physiological process of skin healing (Sullivan et al., 2001). In addition, pigs can cause larger TBSA burns than murine, and the hypermetabolic response after burns is more similar to that of human patients. These advantages make pigs gradually become the first choice for burn animal models. However, the pig burn model is still much lower than that of the mouse and rat (Abdullahi et al., 2014). On the one hand, it may be related to the size of the animals. Establishing a pig burn model has a greater risk, which may bring the risk of burn to the experimenter. On the other hand, from an economic point of view, its economic cost is much higher than that of a murine model. Studies have shown that purchasing and raising a 30-day-old pig costs nearly $800, while rats and mice cost less than $100 (Abdullahi et al., 2014). In addition, the gestation period of pigs is around 100 days, while the murine is usually 20 days, which is also an important reason why the cost of mouse models is much lower than that of pig models. Currently, animal studies on the intestinal microbiome after severe burn are few, especially in large animals. Most experiments on intestinal microbiome after severe burn were conducted with the murine model. Despite the similarity of the intestinal microbiome of mice to that of humans at the phylum level, a huge difference is observed at the species level (Hugenholtz and de Vos, 2018; Nagpal et al., 2018). Moreover, the human intestinal microbiome is generally collected from feces, whereas mice are collected from caecal. The difference in the sampling location also interferes with the results. However, the dominant microbiome in human and murine intestines is similar, and the effects of the microbiome on intestinal function are conservative. Therefore, the murine model is still valuable for studying gut microbiome after severe burn. Beckmann et al. (2018) used CF-1 mice to establish a full-thickness scald injury model. Compared with the control group, the injury model demonstrated an obvious increase in the abundance of gram-negative bacteria, such as *Proteobacteria*, *Deferribacteres* and *Bacteroidetes*, which initially had low abundance in healthy intestines. The number of species from the phylum *Proteobacteria* increased significantly, especially in the subfamily *Enterobacteriaceae*, which is consistent with the results of previous research. The *Enterobacteriaceae* family contains many opportunistic pathogens common in patients with sepsis, such as *Escherichia*, *Klebsiella* and *Proteus*. These bacteria may aggravate systemic inflammation after burns. However, no studies have reported which individual strains of these bacteria play a significant role. The growth of many pathogenic bacteria is also accompanied by a decrease in the number of beneficial bacteria, weakening the barrier function of the intestinal tract. Studies have found that the abundance of bacteria producing SCFAs in the intestine of burnt mice decreased, resulting in a decrease in the level of SCFAs in the intestines and a decrease in intestinal pH (Kuethe et al., 2016). Huang et al. (2017) used Sprague-Dawley rats to establish a burn injury model. They revealed a significant reduction in the number of butyrate-producing bacteria, such as *Clostridium IV* and *Clostridium XIV* and decreased levels of *Lactobacillus*. *Lactobacillus* plays an essential role in maintaining the SCFA concentration in the intestines. The metabolite lactate produced by *Lactobacillus* can be utilised as a substrate by other bacteria to produce butyrate easily. Beckmann et al. (2018) also found that most butyrate-producing bacteria in the intestinal tract decreased after burns, such as *Lactobacillaceae* and *Clostridiaceae* families. Most of these bacteria belonged to the phylum Firmicutes. Furthermore, Feng et al. (2019) reported that the number of *Lactobacillus* greatly reduced after burns, significantly correlated with SCFA levels and negatively correlated with the abundance of *Escherichia-Shigella*. Butyrate is a kind of SCFA with an essential function in the intestines. It can provide energy for intestinal cells for metabolism and regulate T cell function and immune response. Studies have demonstrated that butyrate can reduce T-cell death through an acid sphingomyelinase-dependent mechanism (Rice et al., 2017). T-cell depletion is crucial in burn-induced immunosuppression and increased susceptibility to opportunistic infection. In the murine model, Kuethe et al. (2016) found that the transplantation of faecal microbiota containing butyrate-producing bacteria can improve colonic permeability caused by burning. Therefore, fecal transplantation may be a new therapy to restore colon health after burns. These results also suggest that butyrate and its producing bacteria may be potential targets for treating burns.

Some confounding variables, such as alcohol usage and advanced age, can complicate the condition of patients with severe burn. Alcohol abuse has become a global problem. Excessive drinking is an increased risk factor for traumatic injury (Smith et al., 1999). Data show that about half of the burn inpatients can detect excessive alcohol levels in their serum upon admission (Silver et al., 2008). Many studies have shown that the combination of alcohol and burns can lead to more serious outcomes, resulting in longer hospital stays, increased susceptibility to sepsis, multiple organ failure and death, and higher surgical rates (Silver et al., 2008; Li et al., 2015). Long-term drinking can lead to an imbalance of the intestinal microbiome and increased endotoxin levels (Mutlu et al., 2009; Mutlu et al., 2012). In addition, the combined injury of alcohol and burn can also lead to increased intestinal permeability, which may be related to the increase of *Enterobacteriaceae* abundance and the decrease of claudins and mucins expression after injury (Hammer et al., 2016). In general, alcohol exposure can aggravate the injury caused by burns, which may be related to the changes in intestinal microorganisms, which is worthy of our in-depth study. The proportion of older people (>65 years of age) in the population is increasing. Most of them are accompanied by chronic diseases, which make them frailer than other age groups. Pre-existing frailty may be the main determinant of the worst outcomes in elderly patients after burn (Romanowski et al., 2019). Sarah et al. (Rehou et al., 2019) found that elderly patients with severe burn have a unique acute response (the first 96 hours after burn), characterised by a decrease in cardiac function and blood pressure, resulting in a decrease in organ perfusion and oxygenation. These factors may further accelerate organ dysfunction and increase mortality in elderly patients with severe burn. In the early stage, elderly burn patients will produce delayed immunity, inhibit the inflammatory response, fail to respond to acute injury rapidly, and change to excessive inflammation in the late stage of burn, which is a manifestation of immune aging of the body. As an acute injury, the burn may deplete the immune reserve of the elderly and lead to immune exhaustion, resulting in higher mortality (Stanojcic et al., 2016). Additionally, the composition of the intestinal microbiome changes with age, such as the decrease of microbiome diversity, the transformation of dominant species, and the changes in some beneficial bacteria and metabolic pathways (Woodmansey et al., 2004; Mueller et al., 2006; Biagi et al., 2010). The abundance of *Bacteroides* in intestinal microorganisms of the elderly increased, while *Clostridium* cluster XIVa, *Faecalibacterium prausnitzii* and *Bifidobacterium* decreased significantly (Hopkins et al., 2001; Claesson et al., 2011; Claesson et al., 2012; Odamaki et al., 2016). As a result of normal aging, microbiome dysbiosis is common in the elderly, which may be related to the changes in intestinal microflora (Claesson et al., 2011; Claesson et al., 2012). Burns may aggravate the severity of microbiome dysbiosis in the elderly, worsen the burns’ complications, and affect the patients’ prognosis. Dyamenahalli et al. (2022) found that the alpha diversity of fecal samples of elderly patients with severe burn decreased significantly, with more *Bacteroides*, while the proportion of *Lactobacillus* decreased sharply. The decrease of alpha diversity of intestinal flora may lead to the overgrowth of intestinal pathogens in elderly patients, increasing the risk of infection and leading to poor prognosis. Although *Bacteroides* play an important role in maintaining the beneficial microbial environment of the intestinal tract, when its proportion is out of balance, it may also cause serious harm, especially in this particular group of elderly patients. Elderly patients with various systemic diseases and immunosenescence further limit their ability to resist infection. *Lactobacillus* functions by inducing mucosal antibodies to protect and maintain the intestinal mucosal barrier. The decrease in its proportion may reduce the immunity of elderly patients and increase their susceptibility to infection. Elizabeth et al. (Wheatley et al., 2020) established a scald model in mice, and fecal samples were used to detect the changes in intestinal microflora in aged and young mice after severe burns. The results showed that the abundance of *Enterobacteriaceae* in aged burned mice was lower than that in young burned mice, indicating that the combination of aging and burn may affect the abundance of a certain intestinal flora. In addition, there were more changes in intestinal flora abundance in elderly burned mice (18 genera decreased and 4 genera increased), indicating that aging significantly affected the degree of intestinal ecological imbalance caused by burn. Meanwhile, they also measured the expression of anti-microbial peptide (AMP) in the ileum. The results showed that the expression of AMP in aged burned mice was significantly lower than that in young burned mice, indicating that it is difficult for elderly animals to produce proper and timely AMP response in the intestine to maintain intestinal microbiome homeostasis. These results show that the intestinal microbiome of elderly burn patients has notable changes, which need to be further studied.

## Intestinal mucosal barrier

### Normal gut barrier

The gastrointestinal tract mucosa is a dynamic multi-layered interface that separates the changing external environment from the closely regulated internal milieu. It has various functions to maintain the body healthy, such as regulating the absorption of nutrients and excluding the potential harmful metabolite from the gut lumen. Meanwhile, it forms a barrier to isolating pathogenic microbiome and their metabolites in the intestinal cavity. The gut barrier is the sum of the structure and function of the intestinal tract that can prevent some pathogens and toxins from passing through the intestinal mucosa into other tissues, organs and blood circulation (**Figure 2**). It includes intestinal mucosal epithelium, intestinal mucus, intestinal microbiome, secretory immunoglobulin, intestine-associated lymphoid tissue, gastric acid, bile salt and hormone, which can be divided into mechanical, chemical, immunological and biological barriers (Camilleri et al., 2012).

The mechanical barrier is the generic name for the complete intestinal mucosal epithelial structure, which is closely connected. It is one of the most critical intestinal mucosal barriers, mainly composed of intestinal epithelial cells such as absorptive cells, goblet cells and Paneth cells, and intercellular junctions. Generally, the mechanical barrier can prevent harmful compounds such as bacteria and endotoxins from invading the gut mucosa and entering the bloodstream (Malago, 2015). The junctional complexes composed of TJs, adherens junctions, desmosome and gap junctions connect the intestinal epithelial cells. At the apical of the junctional complexes, TJs form a network of branched closed stripes to restrict the passage of proteins and lipids, thereby regulating intestinal mucosal permeability to small molecules and maintaining cell polarity. Four transmembrane protein families, occludin, claudins, junctional adhesion molecule and tricellulin, are involved in TJ formation. The extracellular domains of these transmembrane proteins form a selective barrier with adjacent cells through homophilic and heterophilic interactions. In the cytoplasm, the intracellular domains of the transmembrane proteins interact with cytoplasmic scaffolding proteins such as zonula occludens (ZO) proteins, which anchor the transmembrane proteins to the actin cytoskeleton. The TJs can limit the solute flux of the paracellular pathway, which is usually more permeable than the transcellular pathway. Therefore, TJs are the key to the rate limit of intestinal mucosal transepithelial transport and the primary determinant of intestinal mucosal permeability. In the process of infecting intestinal cells, some pathogens can target the structural proteins of TJs, destroy the integrity of the intestinal barrier and increase intestinal mucosal permeability (Roxas and Viswanathan, 2018). The chemical barrier is composed of antibacterial substances produced by the resident gut microbiome and chemical substances secreted by gut mucosa, such as mucus, bile, gastric acid, digestive enzymes, mucopolysaccharide, and muramidase, which can inhibit the adherence and colonisation of microbiome (Fink, 2003). The immunological barrier mainly consists of intestinal mucosal lymphoid tissue and secretory IgA produced by plasma cells. In gastrointestinal mucosa, 25% are lymphoid tissue, containing a large number of immunocompetent cells, such as dendritic cells, macrophages, lymphocytes and plasma cells, which play an important role in antigen presentation, mucosal allergic response and inflammatory mediators secretion, which prevent pathogenic antigens from causing damage to the body through cell-mediated and humoral immunity (Berin et al., 2006). The sIgA produced by intestinal associated lymphoid tissue (GALT) can selectively coat Gram-negative bacteria, form antigen-antibody complex, hinder the binding of bacteria to epithelial cell receptors, stimulate intestinal mucus secretion and accelerate the flow of mucus layer, which can effectively prevent bacteria from adhering to the intestinal mucosa. Under trauma, infection, shock and other stress states, GALT was selectively inhibited, and sIgA secretion decreased, which increased the chance of bacterial adhesion and translocation (Ahluwalia et al., 2017). The biological barrier is the resident intestinal microbiome, which can resist the invasion of pathogenic bacteria by forming a biofilm on the intestinal mucosa surface, such as *Lactobacillus* and *Bifidobacterium*. In addition, the resident bacteria can produce short-chain fatty acids and lactic acid to provide energy for intestinal epithelial cells. The resident bacteria and the microspatial structure of the host form an interdependent and interactive microecosystem, which is critical for maintaining the dynamic balance between the host’s immune response and microbiome.

### Changes in gut barrier after burns

As the most pivotal part of the intestinal mucosal barrier, the mechanical barrier plays a vital role in maintaining the homeostasis of the intestinal environment and helping the host to resist pathogenic bacteria. The occurrence and development of many gastrointestinal diseases are closely related to the lack of integrity of the mechanical barrier, and severe burns are no exception. There are many reasons for the damage and dysfunction of the intestinal mucosal mechanical barrier caused by severe burn. Stress response, ischemia, hypoxia, proinflammatory, bacteria and their endotoxins are directly or indirectly involved in the occurrence and development of mechanical barrier damage caused by severe burn. In addition, the destruction of the intestinal mechanical barrier is closely related to shock after burn, hypermetabolism response, immune disturbance, sepsis and multiple organ dysfunction syndromes. These factors may destroy the intestinal mechanical barrier by affecting the expression of TJs protein or causing its relocalisation (Demaude et al., 2006; Graham et al., 2006; Wang et al., 2006; Li et al., 2009; Qin et al., 2009). Feng et al. (2019) found in the burned mice model that the expression of ZO-1, occludin, claudin-1 and claudin-2, the crucial members of intestinal epithelial cells, decreased and showed morphological destruction 1 day after burn, which was consistent with the change of intestinal permeability after burn. Early et al. (Earley et al., 2015) detected intestinal permeability of burn and sham group mice one day after the burn or sham injury procedure by intragastric administration of FITC-dextran. The results showed that the concentration of FITC-dextran in plasma increased 1 day after burn in the burn group, while any changes were observed in the control group within 3 days. In addition, by detecting the expression of TJ protein claudin-4 and claudin-8, TJ protein’s expression level decreased significantly in the burn group. These results showed that the expression of TJ protein in intestinal epithelial cells decreased after burn, the TJs between cells could not be located, and were closely related to the change of intestinal permeability. Dietch et al. (Deitch, 1990) detected the intestinal permeability of burn patients by using lactulose and mannitol as permeability markers. The results showed that the absorption rate of lactulose and mannitol in severe burn patients was much higher than that in the control group within 24 hours after injury, indicating that intestinal permeability increased in a short time after severe burn. Interestingly, Huang et al. (2018) found that the intestinal permeability of 30%TBSA III degree scalded mice increased significantly at 2 hours after scald and peaked at about 4-6 hours after scald, while the histological structure of intestinal mucosa did not change significantly at 2 hours after scald. It shows that after severe burn, although the increase of intestinal permeability is closely related to the change of mucosal tissue structure, the increase of intestinal permeability is not synchronised with the damage of mucosal tissue structure. The increased intestinal permeability caused by severe burn may be earlier than the histological changes of mucosa. This phenomenon may be mainly due to the dysfunction of the intestinal epithelial TJ barrier. However, the damage to the mucosal structure will undoubtedly aggravate the increased permeability. Once intestinal permeability increases, intestinal bacteria and endotoxins break through the broken intestinal barrier and then quickly spread to distant organs, such as the liver, lungs, and spleen, and even to the bloodstream through the portal vein or lymphatic system. Xiao et al. (Xiao, 2008) traced the endotoxin in severe burn rats with (Zhang et al., 2020)I labelling. It was found that the content of endotoxin in 15min increased at the portal vein and reached the peak at 6 h after burn. Jones et al. (1990) found bacterial translocation in mesenteric lymph nodes in 30%TBSA scalded rats on the first day after burn. Bacteria could continue to translocate to abdominal organs and the bloodstream after infection.

**Figure 3:**
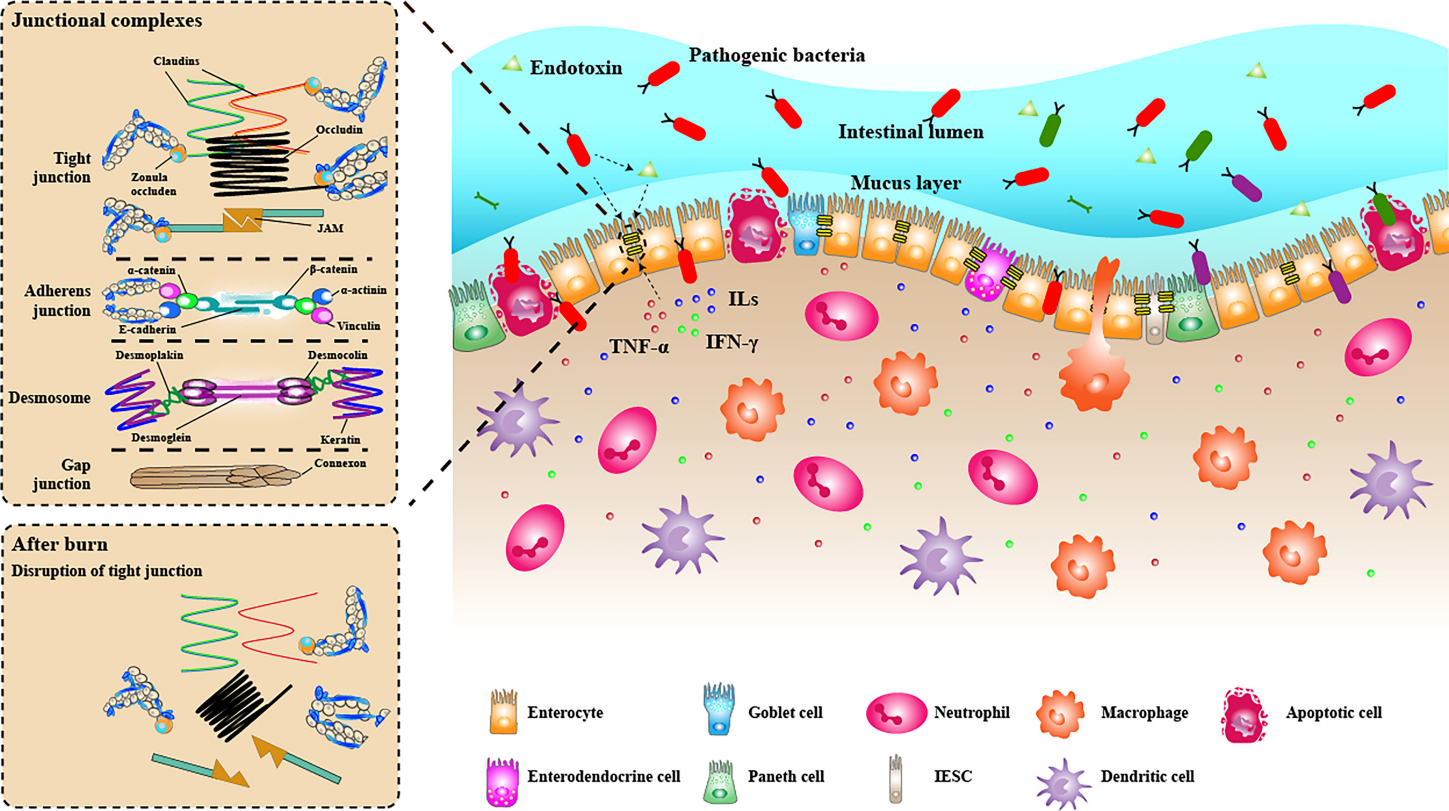
The intestinal mucosal barrier under the condition of burn injury. The intestinal mucosal barrier was seriously damaged under burn conditions. The junction structure between intestinal epithelial cells was destroyed, some intestinal epithelial cells were apoptotic, and the mechanical barrier lost its integrity. The mucus layer becomes thinner or even disappears. Many inflammatory cell (neutrophils, macrophages, dendritic cells) infiltrated the lamina propria, releasing proinflammatory cytokines (TNF-α, ILs, IFN-γ), further destroying the intestinal barrier. The diversity of bacteria in the intestinal lumen decreased significantly, while pathogenic bacteria had excessive growth. Some bacteria can penetrate the broken intestinal barrier, invade the lamina propria and carry endotoxin translocation into the bloodstream.

The interaction between the intestinal microbiome and intestinal epithelial cells can affect the intestinal barrier function, gradually attracting people’s attention in recent years. Especially in some chronic gastrointestinal inflammatory diseases, pathogens and symbiotic bacteria have been widely studied. Beneficial symbiotic bacteria can enhance intestinal mucosal barrier function by reducing intestinal barrier permeability and increasing the expression of TJ proteins (Anderson et al., 2010; Martens et al., 2018). Bacterial metabolites such as butyrate, acetate, and indole also play an essential role in enhancing intestinal barrier function (Hamer et al., 2008; Bansal et al., 2010; Fukuda et al., 2011). On the other hand, pathogens can target TJ proteins, thus destroying the intestinal mucosal barrier. Among the microbe, enteropathogenic *Escherichia coli* (EPEC) is the most studied in regulating gut barrier function. EPEC has a type III secretory system (T3SS), which can secrete a variety of effector proteins, such as EspF, MAP and EspG, and inject them into intestinal epithelial cells, resulting in cytoskeleton collapse and relocalisation of TJ proteins by modifying or blocking the signal pathways to synthesise TJ proteins (McNamara et al., 2001; Ugalde-Silva et al., 2016). ML-9, a specific chemical inhibitor of MLCK, can prevent intestinal barrier dysfunction caused by EPEC infection, indicating that MLCK-MLC phosphorylation pathway may be the signal pathway of intestinal barrier dysfunction caused by EPEC (Yuhan et al., 1997). NF-κB and PKC pathways are also suggested to be involved in breaking the gut barrier induced by EPEC infection (Yuhan et al., 1997). Additionally, enteroinvasive *Escherichia coli* (EIEC) can also cause decreased expression of TJ proteins occludin, ZO-1,claudin-1 and JAM-1 and relocalisation, which in turn leads to intestinal barrier dysfunction (Qin et al., 2009). Endotoxin has been proven to destroy the intestinal barrier, mainly through the following two ways: 1, directly injure intestinal epithelial cells; 2, induce cells to produce proinflammatory mediators to destroy the intestinal barrier, such as TNF-α and IL-1β. The endotoxin is reported to destroy the intestinal epithelial barrier of rats by relying on MLCK, and ML-7, a specific inhibitor of MLCK, can reduce intestinal epithelial dysfunction caused by endotoxin (Moriez et al., 2005). In addition, endotoxin can cause relocalisation of ZO-1 and claudin-1 and reduce the expression of ZO-1 through c-Src-dependent mechanism that involves toll-like receptor-4 (TLR4) and LPS binding protein (LBP), thus destroying tight junctions (Sheth et al., 2007). The above studies have shown that pathogenic bacteria and endotoxin can destroy the intestinal barrier and increase intestinal permeability. However, there are few studies on the effect of intestinal microbiome changes on the integrity of the intestinal mucosal barrier after severe burn. The current research evidence can only show that intestinal microbiome dysbiosis occurs after severe burn. The increase in the number of some pathogenic bacteria, such as *Enterobacteriaceae*, may aggravate the destruction of the intestinal barrier and promote the transfer of bacteria and their products from the intestinal tract to the bloodstream and other organs (**Figure 3**). Further research in this area can provide new targets for treating sepsis, multiple organ failure and other complications caused by severe burn.

## Strategies and potential of microbial therapy

The composition and function of the intestinal microbiome usually change significantly after severe burn, affecting the disease’s progress and prognosis. In the early stage, intestinal microbiome diversity decreased significantly after severe burn. Among them, obligate anaerobes and *Bifidobacterium* gradually reduced, while the abundance of opportunistic pathogens such as *Escherichia coli* and *Enterococci* increased significantly. These changes are closely related to the subsequent destruction of the intestinal mucosal barrier, intestinal bacterial translocation and secondary systemic infection. After treatment intervention, the intestinal microbiome began to remodelling in the late stage after severe burn, and beneficial bacteria significantly increased. At the same time, most of the opportunistic pathogens decreased and gradually returned to normal (Shimizu et al., 2015; Huang et al., 2017; Wang et al., 2017; Feng et al., 2019). These results suggest that intestinal microbes may play an essential role in the whole process of severe burn injuries. The regulation of intestinal flora may be a favourable direction for burn treatment. A close relationship exists between intestinal microorganisms and the host, affecting the intestinal tract’s physiological and pathological states. Therefore, after years of research, disease treatment by regulating intestinal microbiota has been proven to be a potential method. Current research focuses on implementing various strategies for intestinal microbiota to manage or prevent complications in some patients with critical illnesses, such as sepsis, urinary tract infections and pneumonia (Adelman et al., 2020). The main clinical approaches used to modify intestinal microbiota are as follows: 1, the use of antibiotics or antifungal agents can consume too many members of the intestinal microbiota or reduce the overall microbial load; 2, Regulate the composition and function of intestinal microflora through dietary control or supplement of live microorganisms (single or mixed species) (Durack and Lynch, 2019). Recently, microflora-based treatment has gradually emerged in use in infectious and gastrointestinal diseases, such as faecal microflora transplantation (FMT), which has promising results.

Selective digestive decontamination (SDD) is one of the few intervention measures in intensive care medicine that can improve the survival rate of patients. The advanced administration of antibiotics not absorbed by the digestive tract can kill the potentially pathogenic bacteria in the intestinal tract without affecting the anaerobes in the intestinal mucosa to reduce the translocation of intestinal flora. Previous studies have presented that sepsis caused by severe diseases can be prevented by selective decontamination of the oral cavity or digestive tract with antibiotics (de Smet et al., 2009; Huttner et al., 2013). Studies have reported that in the intensive care unit (ICU), patients receiving SDD have significantly less bacteraemia caused by *Staphylococcus aureus* and *Enterobacteriaceae* than patients receiving standard treatment (de Smet et al., 2009). A systematic review of studies on SDD and non-absorbable enteral antibiotics (EA) in patients with severe burn revealed that the SDD or EA treated group had a lower incidence of *Enterobacteriaceae* in the bloodstream than in the placebo and non-treatment groups. And the incidence of pneumonia decreased only in the group using SDD (Rubio-Regidor et al., 2018). To our knowledge, no study has shown that SDD causes antibiotic resistance, but SDD can promote an increase in methicillin-resistant *Staphylococcus aureus* (Vincent and Jacobs, 2011). Although the studies have found that SDD is safe in patients with critical illness, SDD may lead to antibiotic resistance (such as increasing incidence of methicillin-resistant *Staphylococcus aureus*) and chronic destruction of the intestinal microflora due to requires long-term and extensive use of antibiotics (Deitch et al., 1985; Feng et al., 2019). Therefore, for the use of SDD, further studies of the complications after antibiotic administration are needed to determine whether it can bring more benefits than disadvantages to patients.

With the expansion of microbial research, microbial-based intervention as a new therapy has gradually become the research focus. Probiotic supplementation to prevent and slow down the intestinal mucosal barrier damage caused by severe diseases is expected to become a new treatment strategy. Probiotics are beneficial bacteria that colonise and alter the flora composition of a particular part of the host’s body. Probiotics are either single or well-defined mixed microorganisms which can promote nutrient absorption and maintain intestinal health by regulating the immune function of host mucosa and system or regulating the balance of intestinal flora. Intestinal probiotics previously studied are *Bifidobacterium*, which has been confirmed to promote gastrointestinal health and relieve intestinal symptoms such as intestinal stress syndrome and diarrhoea caused by antibiotics (Tojo et al., 2014). *Bifidobacterium* is one of the essential SCFAs-producing bacteria, and many studies have revealed that the abundance is significantly reduced in the intestine tract of a patient with severe burn. Although *Bifidobacterium* has been widely used in intestinal inflammatory diseases, its usage in patients with severe burn is still in the research stage. The results of a study on the use of *Bifidobacterium* in rats with burns revealed that it could reduce intestinal mucosal injury and bacterial translocation (Wang et al., 2006). In addition to *Bifidobacterium*, it is reported that other butyrate-producing bacteria have specific therapeutic potential. A recent study indicated that the oral administration of *Clostridium butyricum* (a class of butyrate-producing bacteria) in mice with burns could increase intestinal butyrate levels, decrease the expression of TNF-α and IL-6, inhibit intestinal injury and increase intestinal permeability (Zhang et al., 2020). These results revealed that probiotic supplementation to target butyrate production for treating intestinal damage caused by severe burn would be a promising research field. Probiotic therapy presents a positive therapeutic effect, but it has some limitations. A study evaluated the impact of prophylactic probiotics on intestinal microbial diversity and function in adult patients with burn. It revealed that after taking *Lactobacillus acidophilus* and *Lactobacillus rhamnosus*, patients developed more diarrhoea and malabsorption symptoms within 1–2 weeks of treatment. However, no significant difference was found in the infection rate and sepsis rate of *Clostridium difficile* infection (CDI), indicating that prophylactic probiotic supplementation in patients with burns is not related to the improvement of prognosis. It may be associated with an increased incidence of diarrhoea and malabsorption (Fleming et al., 2019). Therefore, the effect of probiotic supplementation in patients with severe burn needs to be further studied to understand the mechanism of microbiota as a target for treatment.

FMT, therapy with a long history that can reconstruct intestinal flora, has received increasing attention. FMT involves transplanting functional microbiota from the faeces of healthy people into the gut of patients, thereby regulating intestinal flora imbalance, rebuilding the intestinal micro-ecosystem with normal function and helping in treating intestinal and extraintestinal diseases. Unlike using one or a few probiotics, FMT can colonise the whole donor intestinal microbiome in the recipient intestinal tract, recombine the recipient intestinal microbiota and regulate the recipient intestinal function through various mechanisms, such as SCFA production and immune regulation. Thus, it has the potential to treat multiple intestinal diseases (Adelman et al., 2020). FMT has shown a good effect in treating dysbiosis caused by CDI, which has become a well-established treatment. FMT may restore the normal gut microbiota composition of the host through direct and indirect pathways to achieve therapeutic effects (Khoruts and Sadowsky, 2016; Ooijevaar et al., 2019). On the one hand, the microbiota of the recipient can directly compete with host pathogenic microorganisms for nutrients and colonisation resources, interfere with their virulence factors, and even kill them directly, thereby enhancing the colonisation resistance of the host gut. In addition, FMT can also directly transplant microbial metabolites into the host intestine, such as SCFAs, bile acids, and bacteriocins (peptides with narrow-spectrum anti-microbial properties produced by bacteria), and these metabolites have a certain inhibitory effect on pathogenic microorganisms. The bile acids are divided into primary bile acids and secondary bile acids. Primary bile acids can promote the germination of *Clostridium difficile*, while secondary bile acids show inhibitory effects (Thanissery et al., 2017). Dysbiosis caused by CDI can affect bile acid metabolism, reducing the conversion of primary bile acids to secondary bile acids. Secondary bile acids can be provided directly in FMT to improve this process. On the other hand, FMT can indirectly enhance the host’s physiological and immune defence capabilities. Activating various immune mechanisms in the host and restoring bile acid metabolism and SCFA metabolism inhibits the germination, growth and sporulation of pathogenic bacteria. Additionally, FMT may promote intestinal mucosa regeneration through tonic signals and the production of mucin and anti-microbial peptides, thereby repairing the damaged intestinal barrier. Although FMT has been proven effective in treating recurrent drug-resistant CDI, it has not presented a similar success rate in some chronic intestinal inflammatory diseases, and the clinical remission stage is more difficult to predict. Among the five cases of FMT-treated sepsis, five patients had stayed in the ICU for a long time because of complications such as respiratory failure, multiple drug-resistant bacterial infections and multiple organ dysfunctions. Among them, after receiving FMT treatment, the organ function and survival rate improved in four patients. Before FMT treatment, the intestinal microbiota of patients was characterised by the dominance of bacteria. In contrast, the intestinal microflora was similar to the donor faeces after FMT treatment, and the abundance of symbiotic bacteria increased (Li et al., 2014; Li et al., 2015; Wei et al., 2016). The acute stage of severe burn patients is often accompanied by multiple organ failure, bacterial infection and other complications. These clinical case reports have considerable guiding significance for treating severe burn. In animal research, Kuethe et al. (2016) prepared FMT from the caecal contents of healthy mice and performed intragastric administration in mice with severe burns. Then, they found that on the sixth day after burns, the integrity of the intestinal mucosa of the mice was restored, and the bacterial imbalance was relieved. The above studies have suggested that FMT may be a feasible method for treating severe burn, but more studies are needed to optimise this treatment further.

Current studies have demonstrated that probiotic supplementation and FMT may be the most beneficial options for treating patients with severe burn (Corcione et al., 2020). However, with advances in microbiome research, we have every reason to expect more accurate and comprehensive multispecies microbial therapies or dietary interventions that target specific individuals or microorganisms.

## Conclusion

In this review, we discussed the changes in the intestinal microbiome after severe burn and their effects on the intestinal mucosal barrier and described microbial therapy. Generally, the role of the changes in post-burn intestinal microflora in disease progression still needs further exploration. Recently, with the wide application of high-through sequencing technology in microbiology, some studies have clarified the changes in intestinal bacterial species and overall function in each stage of severe burn. However, no studies have investigated the molecular mechanism of microbiota changes on intestinal immune barrier function after severe burn. After severe burn injuries, intestinal microorganisms are not balanced, opportunistic bacteria grow in abundance, the abundance of beneficial bacteria decreases, and levels of some bacterial metabolites (such as butyrate) that protect the intestinal mucosal barrier are reduced. Furthermore, disrupting the intestinal mucosal barrier leads to bacterial translocation and bacteraemia, resulting in abnormal systemic immune response and multiple organ failure. These are the functions of the intestinal microbiome in the pathophysiological changes in patients analysed in the current study. However, the specific molecular mechanisms and linkages between these changes need further investigation. With the initial success of probiotic supplementation and FMT in some intestinal inflammatory diseases, they are expected to become potential strategies for treating follow-up complications caused by intestinal changes in patients with severe burn. Microbiome studies in intestinal diseases are mainly focused on inflammatory diseases and tumours. Therefore, further studies are needed in burn models and clinical cases to provide rich survey data for developing new therapeutic targets or drug preparations.

## Author contributions

ZH, YH, QW, and BJ wrote the manuscript; JC, ZT, YC, HL, MH, LQ, and LL made the figures and edited the manuscript; QW and BJ conceived of and supervised the work. All authors have read and agreed to the published version of the manuscript.

## Funding

This work was supported by the Natural Science Foundation of Guangdong Province (grant numbers 2019A1515010408), and the Medical Science and Technology Research Fund Project of Guangdong Province (grant numbers A2018444).

## Conflict of interest

The authors declare that the research was conducted in the absence of any commercial or financial relationships that could be construed as a potential conflict of interest.

## Publisher’s note

All claims expressed in this article are solely those of the authors and do not necessarily represent those of their affiliated organizations, or those of the publisher, the editors and the reviewers. Any product that may be evaluated in this article, or claim that may be made by its manufacturer, is not guaranteed or endorsed by the publisher.
